# Exogenous Ochronosis: Clinicopathological Correlation in Indian Patients and the Practical Applicability of the Dogliotti and Phillips Classification Systems

**DOI:** 10.7759/cureus.49620

**Published:** 2023-11-29

**Authors:** C Divyalakshmi, Subashini Selvadurairaj, Pavithra Sukumaran, Sruthi Ganesh, Renita Lourdhurajan

**Affiliations:** 1 Aesthetic Dermatology, Render Skin and Hair, Chennai, IND

**Keywords:** exogenous ochronosis, dermoscopy, melasma, facial melanosis, hydroquinone

## Abstract

Exogenous ochronosis (EO) results as a complication of long-term usage of skin lightening creams containing hydroquinone or other bleaching agents. Duration of use and concentration of hydroquinone in the product are noted to be key factors that decide the occurrence of EO. With more cases being reported globally, current classification systems lack practical applicability and may not be adequate for detecting early cases. Dermoscopy and clinicopathological correlation are very important for early diagnosis of EO to avoid undue overuse of hydroquinone leading to further deterioration of pigmentation.

We report a series of six patients in one year with EO with the minimum duration of use of hydroquinone being three months to the development of ochronosis. The most common strength of hydroquinone used was 2%, documented in 5/6 cases. Three out of six patients (50%) had discordant findings according to the Dogliotti classification, while four out of six patients (66.7%) had discordant findings according to the Phillips classification.

Our findings suggest that EO can occur with a shorter duration of hydroquinone use, even at lower percentage strengths. We propose that it may be more useful to accept the clinical presentation supported by dermoscopic features as adequate actionable findings, consider all the histopathological stages as warning signs of ochronosis or impending ochronosis, and terminate the use of hydroquinone in such patients.

## Introduction

Exogenous ochronosis (EO) remains relevant in settings where hydroquinone is frequently used to manage hyperpigmentation [1]. Earlier considered a complication of topical hydroquinone use only over the long term [2], some studies have pointed to a shorter period of hydroquinone usage leading up to a diagnosis of ochronosis. Second, from few and far between case reports, the number of EOs has been increasingly reported in recent days [3,4]. So far, the only classification systems with a clinicopathological correlation in use for grading EO are those proposed by Phillips and Dogliotti, despite some evidence of their lack of practical applicability [3]. Three clinical stages are described for EO according to Dogliotti, the first is erythema and mild pigmentation; the second is hyperpigmentation, milia, and mild atrophy; and the third stage presents with papulonodular lesions [5]. Unlike endogenous ochronosis, there is no systemic involvement. Considering the importance of early diagnosis, which can facilitate prompt termination of hydroquinone usage [6], the significance of early-stage histological findings is very relevant for preventing the worsening of the pigmentation. Herein, we present a series of six patients with EO diagnosed within a period of one year and discuss the relevance of clinicopathological grading systems in this group.

## Materials and methods

We report a case series of six cases of EO, who visited our center over a period of one year, identified during our annual chart review between February 2022 and February 2023. We recorded a total of six cases during this period, based on a histopathological diagnosis of EO. Each of them had presented with progressively worsening hyperpigmented patches on the face, with a history of using multiple topical applications over variable time periods for the same.

The history, clinical features, dermoscopic findings, and histopathological features were reviewed from their medical records, compiled, and presented in this series. Specifically in history, the duration of the condition, the use of topical agents for skin lightening, specific use of hydroquinone, and mitigation or worsening of symptoms after use of such agents were obtained from the medical record. The percentage of hydroquinone use, and duration of use, were deduced from any previous prescriptions from earlier medical providers that were recorded as part of the patient’s past history, if the same was available.

On presentation, a clinical assessment and dermoscopy were done, and where findings were suggestive of ochronosis, a biopsy was performed to obtain a confirmatory histopathological diagnosis of ochronosis. The clinical features recorded included patchy deep brown hyperpigmented macules over the face. Dermoscopy was done by Dermlite DL4 (10x; DermLite LLC, San Juan Capistrano, CA), and a diagnosis of EO was considered based on the presence of multiple dark brown globules with worm-like structures and focal areas of follicular obliteration.

The site of the biopsy was guided by dermoscopy as being the most appropriate to provide the best yield. A diagnosis of EO was based on histopathological findings of scattered melanophages in the papillary dermis, thinning and basophilia of collagen fibrils, and mild brownish discoloration of collagen bundles in the upper reticular dermis. Clinical descriptions of the lesions and associated clinical photographs were available for all cases. Dermoscopic images were available for five out of six cases. Histopathology slides were available for review in all six cases. The severity of EO was graded using the Dogliotti and Phillips classification systems [1,3], and the concordances were noted.

According to the Dogliotti classification system, clinical stages are classified as follows, stage 1 for erythema and mild hyperpigmentation; stage 2 denotes progressive hyperpigmentation, pigmented colloid milium, caviar-like lesions, and scanty atrophy; and stage 3 indicates the presence of papulonodular or sarcoid-like lesions. Histopathological stages 1 and 2 are combined into a single stage (stage 1/2), where the findings include epidermal changes and blocks of ochronotic pigment in the dermis, and ochronotic pigments with sarcoid-like granulomas are classified as stage 3 [1,3].

For the Phillips classification system, clinical stages are noted as mild for features of coarsening and darkening of the skin, moderate for large black papules with intervening normal skin, and severe for larger, coalescing lesions with relatively dark caviar-like papules. Histopathological grading is based on changes in collagen fibrils. Grade 1 denotes basophilic changes of collagen fibrils, grade 2 is characterized by an increased diameter and basophilia of collagen fibers, grade 3 includes yellow-brown ochronotic pigment within collagen fibrils, and grade 4 signifies dissolution of collagen with eosinophilic miliary materials [1,3].

## Results

**Figure 1:**
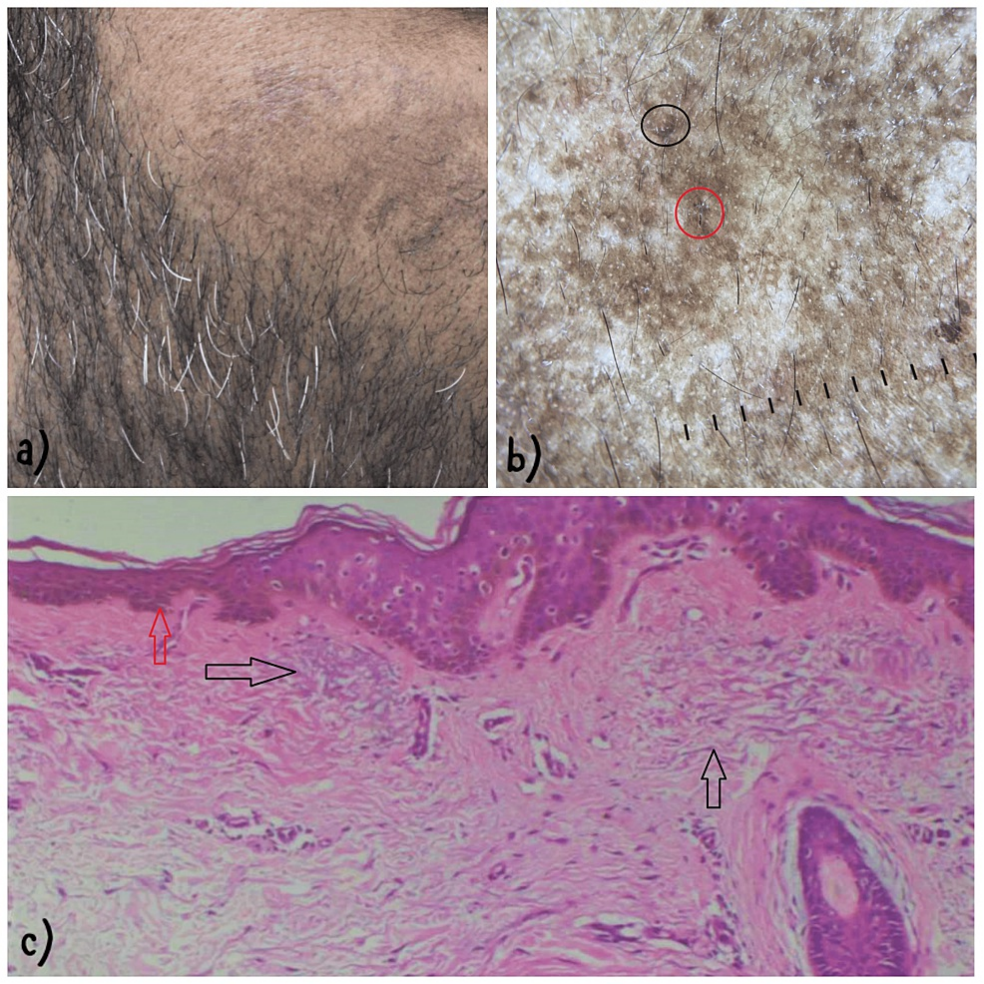
Clinico-dermoscopic and pathological concordance according to both Dogliotti and Phillips classification systems. a) Clinical image showing patchy hyperpigmented macules on both cheeks on the right cheek in a 47-year-old male. b) Dermoscopy showing accentuation of pseudoreticular pattern of pigment network. Dark brown globules with worm-like structures (black circle) and focal areas of follicular obliteration (red circle). c) Photomicrograph (H & E, 10x) showing basal layer hyperpigmentation (red arrow). Basophilic changes in collagen bundles (black arrow) in the upper reticular dermis with mild brownish discoloration of collagen fibrils.

**Figure 2:**
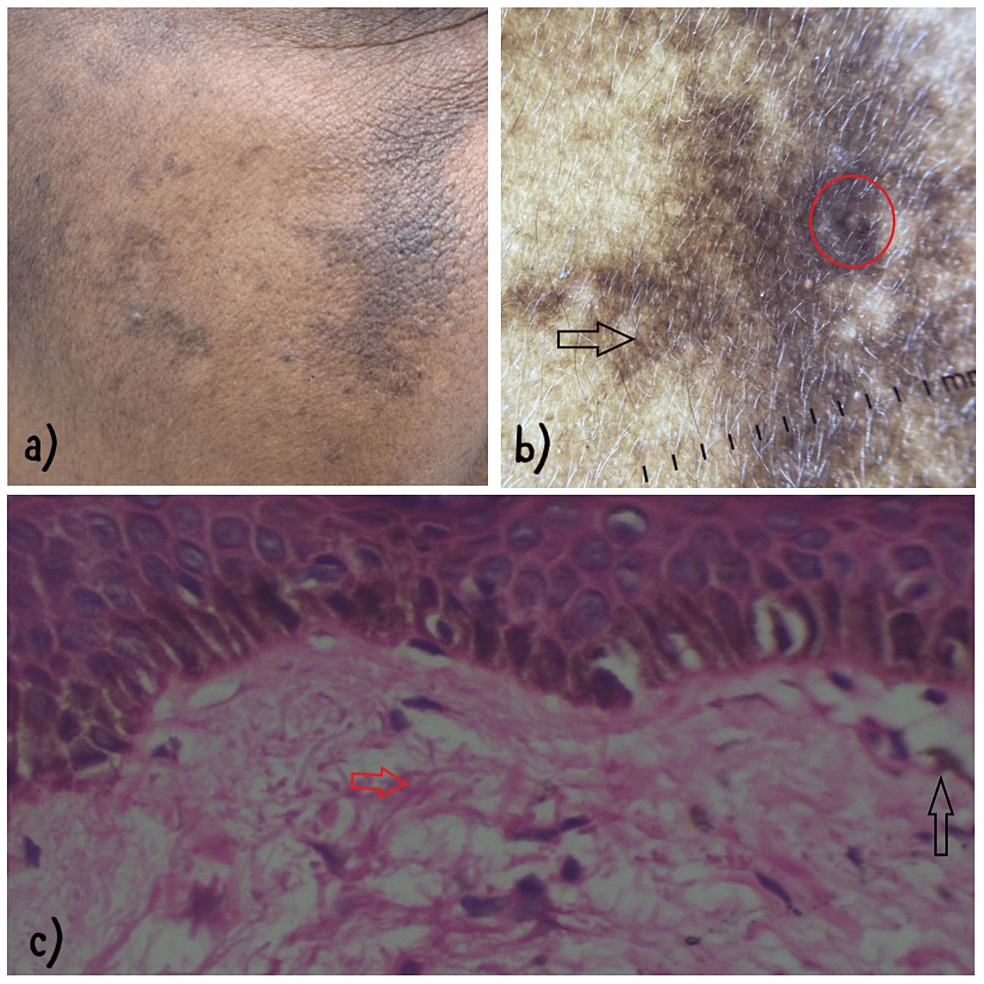
Clinico-dermoscopic and pathological discordance according to both Dogliotti and Phillips classification systems. a) Clinical image showing patchy slate grey- to deep-brown pigmented macules on the right cheek in a 47-year-old female. b) Dermoscopy showing brown amorphous areas with a few areas of follicular obliteration (black arrow), caviar-like coarse brown deposits (red circle). c) Photomicrograph (H & E, 40x) showing hyperpigmentation of the basal layer of the epidermis. Scattered melanophages in the papillary dermis. Areas of thinning of collagen fibrils (red arrow). Mild brownish discoloration of collagen bundles in the upper reticular dermis (black arrow).

Out of the six cases, there were five females and one male. Five out of six cases reported a definite usage of 2% hydroquinone-containing creams, and the percentage of hydroquinone could not be ascertained in one individual. The minimum duration of use was three months in one patient prior to presentation, while two reported using hydroquinone for six months and the other two for about a year, before noticing a deepening of pigmentation. Almost all of them had been using the creams over the counter, beyond the prescribed period. Relevant historical data are shown in Table 1. The clinical, dermoscopic, and histopathological features and their concordance with Dogliotti and Phillips classification systems are presented in Table 2. Figures 1-2 show clinical, dermoscopic, and histopathological findings in two of our cases. Three out of the six patients (50%) had discordant findings according to the Dogliotti classification, while four out of the six patients (66.7%) had discordant findings according to the Phillips classification.

**Table 1:** Historical data of the study subjects.

Study Subjects	Age	Gender	Indication for which hydroquinone was started	Duration of hydroquinone use before onset/worsening of pigmentation	Percentage of hydroquinone	Prescription/unsupervised use
A	47	F	Melasma	Three months	2%	Prescription
B	47	M	Not available	Not available	-	Over-the-counter fairness creams, on and off, for more than 15 years
C	35	F	Melasma	Six months	2%	Prescription + unsupervised use
D	35	F	Facial melanosis (not specified)	One year	2%	Prescription + unsupervised use
E	45	F	Facial melanosis (not specified)	Six months	2%	Prescription + unsupervised use
F	52	F	Facial melanosis (not specified)	One year	2%	Prescription + unsupervised use

**Table 2:** Clinico-dermoscopic and pathological correlation according to both Dogliotti and Phillips classification systems in the study subjects.

Study subjects	A	B	C	D	E	F
Clinical features	Patchy slate grey to deep brown pigmented macules on both cheeks, forehead, and the nose.	Patchy hyperpigmented macules on both cheeks.	Patchy brownish pigmentation on bilateral malar areas.	Brownish patchy pigmentation on the forehead, temples, and lateral cheeks.	Hyperpigmented macules over both cheeks and the nose.	Diffuse brownish pigmentation over the forehead, cheeks, and auricles.
Findings on dermoscopy	Brown amorphous areas with a few areas of follicular obliteration.	Accentuation of pseudoreticular pattern of pigment network. Dark brown globules with worm-like structures and focal areas of follicular obliteration.	Accentuation of pigment network pattern. Areas of follicular obliteration and pinpoint white dots.	Accentuation of pigment network pattern. Occasional blue-black deposits obliterate the follicular openings.	Accentuation of pigment network pattern. Dark brown to blue-black colored caviar-like deposits obliterate the follicular openings.	Accentuation of pseudoreticular pattern of pigment network. Arciform and curvilinear dark brown deposits around follicular openings, with obliteration of few openings.
Biopsy findings	Hyperpigmentation of the basal layer of the epidermis. Scattered melanophages in the papillary dermis. Brownish discoloration of collagen bundles in the upper reticular dermis.	Brownish-colored collagen bundles in the upper reticular dermis. Mild hyperplasia and hyperpigmentation in the overlying epidermis.	A few yellow-brown colored collagen bundles in the papillary dermis.	Hyperpigmentation of the basal layer of the epidermis. Numerous scattered melanophages in the papillary dermis. Increased diameter and basophilia of collagen fibers in some areas.	Several scattered yellow-brown coloured collagen bundles in the upper dermis. Mild hyperplasia and mild increase in basal pigmentation in the overlying epidermis.	Numerous yellow-brown colored collagen bundles in the papillary dermis. Thinning of collagen bundles in the upper reticular dermis. Mild hyperplasia in the overlying epidermis.
Dogliotti (clinical classification)	Three (late)	Two	Three (early)	One	Three (early)	Two
Dogliotti (histopathologic classification)	Stages 1-2	Stages 1-2	Stages 1-2	Stages 1-2	Stages 1-2	Stages 1-2
Concordance	Discordant	Concordant	Discordant	Concordant	Discordant	Concordant
Phillips (clinical)	Severe	Moderate	Moderate	Mild	Severe	Mild
Phillips (HPE)	Grade 2	Grade 3	Grade 3	Grade 2	Grade 3	Grade 3
Concordance	Discordant	Concordant	Concordant	Discordant	Discordant	Discordant

## Discussion

EO remains highly relevant in populations with extensive use of skin-lightening agents [7]. The earliest reported case of ochronosis dates back to over 3,000 years, with EO having been identified on the skin from a mummy’s head, unearthed around 200 BC [8]. Formally, ochronosis was first described by Findlay et al., who noted that the rubbing of the product into the skin and strong sun exposure were factors that led to more severe cases of pigmentation. Findlay et al. described ochronosis as pigmentation that occurs "when the melanocytes overcome the effect of bleaching" and because of "oversaturation of the skin with hydroquinone" [9,10]. The largest series in recent times was reported by Lazar et al., with 25 cases of EO, in 10 years [11]. Earlier, Tan [4] reported 15 cases over four years, and four cases were reported in a period of 15 years by Ramia de Cap et al. [3]. In this study, we report a series of six cases in an Indian population.

Duration of hydroquinone use is considered the most important factor for the development of ochronosis [2,12], with EO reported to develop after the extended use of hydroquinone [1,13-15]. Lazar et al. reported that patients used topical hydroquinone for an average of 9.2 years before developing EO [11]. The longest duration in our current case series is significantly shorter, at one year of usage. Interestingly, in the first-ever report of EO from India, the duration of occurrence of EO was reported to be three months [16]. This case report was that of a female farmer, who developed EO after unsupervised application of 4% hydroquinone for more than three months for melasma, similar to a case in our study.

The second most significant contributor to the development of EO is the concentration of hydroquinone used for skin bleaching. Formulations containing 2% or more hydroquinone are considered adequate for the induction of EO [11], although ochronosis has been most commonly reported with the use of 4% hydroquinone [17]. The widely used triple combination (topical corticosteroid/tretinoin/hydroquinone) creams from India contain between 2% and 4% of hydroquinone; however, elsewhere internationally, topical hydroquinone is available in concentrations of up to 10% [18].

Skin type and sun exposure are other known predisposing agents to the development of ochronosis [9-11,17]. It is possible that a high UV index in our geography plays a role in the earlier induction of EO, a factor highlighted by Findlay et al. when describing the earliest reported cases of EO from Africa [9]. Reports also suggested that rubbing, or inunction of the cream into the skin, was a significant factor, but this could not be ascertained in our series [9,10].

Ochronosis can also result from skin-bleaching serums containing resorcinol, phenol, or mercury, although hydroquinone remains the most implicated agent [7]. Based on history, we were able to identify hydroquinone as the inciting agent in the majority of subjects in the current series. Rampant use of triple combination (topical corticosteroid/tretinoin/hydroquinone) for skin bleaching and skin fairness in certain regions of India, the combination of increasing incidence, and faster time to clinical manifestation have the potential to snowball into a much larger caseload of EO than anticipated [18].

The clinical appearance is often the first sign to consider the diagnosis of ochronosis, with coarse skin, stippled macular pigmentation, and occasional areas of normal intervening skin, being seen as the main findings. A history of bleaching cream use or hydroquinone use was the next significant point of suspicion, which was then further strengthened by dermoscopic features, typically occlusion of the eccrine/follicular openings with dark dense pigment and caviar-like pigment deposits. Given that early ochronosis may appear indistinguishable from melasma with only erythema, mild hyperpigmentation, or nonspecific cases such as skin coarseness, dermoscopy is indicated in every case of facial melanosis, especially with a history of topical bleaching cream use [6,19]. We also recommend that dermoscopy be used to guide the site of the biopsy to provide the best yield, as suggested in earlier reports as well [20].

Biopsy is definitely considered the gold standard for the diagnosis of EO; however, as seen above, the clinical severity does not always seem to correlate with the histopathological grade. In our case series, the presence of early collagen fibril changes, which would mean a lower histopathological grade, did not match with a milder clinical presentation. None of the patients had very obvious histopathological findings of ochronosis. Only subtle and early histopathological changes of ochronosis were found, highlighting the fact that a clinicopathological correlation is warranted in such scenarios. Dogliotti's classification seems practically nonapplicable as a classification system, much in agreement with published work [2]. Conversely, the severity of the clinical features did not seem to be adequately captured by the Phillips classification as well (Table 2). Consequently, if clinicopathological correlation is underscored as a requisite for the confirmation of diagnosis, early cases may be missed, and the offending causative agent will continue, leading to further worsening of the pigmentation, which seems to be clinically refractory at this point [3].

We propose that it is more prudent to appreciate the clinical presentation aided by dermoscopic findings along with corroborative histopathological findings (irrespective of the clinical stage) of ochronosis or impending ochronosis at the earliest and terminate the use of hydroquinone on patients. The need for histopathological findings in EO [21,22] should not be allowed to overrule the clinical diagnosis aided by dermoscopy.

## Conclusions

From our case series of six cases in a span of a year, we can conclude that ochronosis is fairly common in populations where hydroquinone and related bleaching cream use is fairly high, on a prescription basis or otherwise. To prevent an increase in the incidence of refractory hyperpigmentation conditions typified by ochronosis, we need more awareness among clinicians about the extent of the issue and a higher level of clinical suspicion even at an earlier stage and a willing dependence on noninvasive diagnostic tools like dermoscopy. When in doubt, histopathological evaluation and subsequent clinico-pathological correlation are required for an early diagnosis of EO.

This is a small and preliminary study to highlight the significance of a high index of suspicion for EO even with a shorter duration of hydroquinone use and the role of clinico-pathological correlation in establishing an early diagnosis. The current classification systems for diagnosing EO are inadequate in prompt diagnosis of EO especially in early cases. However further "ascertain" the practical usability or "disprove" the same, of the already used ochronosis classifications (Phillips and Dogliotti), more case studies are required in the future.
